# The effects of a 2-h trial of high-flow oxygen by nasal cannula versus Venturi mask in immunocompromised patients with hypoxemic acute respiratory failure: a multicenter randomized trial

**DOI:** 10.1186/s13054-015-1097-0

**Published:** 2015-11-02

**Authors:** Virginie Lemiale, Djamel Mokart, Julien Mayaux, Jérôme Lambert, Antoine Rabbat, Alexandre Demoule, Elie Azoulay

**Affiliations:** Medical ICU, Saint Louis Teaching Hospital, AP-HP, 1 avenue Claude Vellefaux, 75010 Paris, France; Medical-Surgical ICU, Institut Paoli Calmettes, 13000 Marseilles, France; Biostatistics Department, Saint Louis Teaching Hospital, AP-HP, Paris, France; Respiratory ICU, Pitié Salpétrière Teaching Hospital, AP-HP, Paris, France; Respiratory ICU, Cochin Teaching Hospital, AP-HP, Paris, France

## Abstract

**Introduction:**

In immunocompromised patients, acute respiratory failure (ARF) is associated with high mortality, particularly when invasive mechanical ventilation (IMV) is required. In patients with severe hypoxemia, high-flow nasal oxygen (HFNO) therapy has been used as an alternative to delivery of oxygen via a Venturi mask. Our objective in the present study was to compare HFNO and Venturi mask oxygen in immunocompromised patients with ARF.

**Methods:**

We conducted a multicenter, parallel-group randomized controlled trial in four intensive care units. Inclusion criteria were hypoxemic ARF and immunosuppression, defined as at least one of the following: solid or hematological malignancy, steroid or other immunosuppressant drug therapy, and HIV infection. Exclusion criteria were hypercapnia, previous IMV, and immediate need for IMV or noninvasive ventilation (NIV). Patients were randomized to 2 h of HFNO or Venturi mask oxygen.

**Results:**

The primary endpoint was a need for IMV or NIV during the 2-h oxygen therapy period. Secondary endpoints were comfort, dyspnea, and thirst, as assessed hourly using a 0–10 visual analogue scale. We randomized 100 consecutive patients, including 84 with malignancies, to HFNO (n = 52) or Venturi mask oxygen (n = 48). During the 2-h study treatment period, 12 patients required IMV or NIV, and we found no significant difference between the two groups (15 % with HFNO and 8 % with the Venturi mask, *P* = 0.36). None of the secondary endpoints differed significantly between the two groups.

**Conclusions:**

In immunocompromised patients with hypoxemic ARF, a 2-h trial with HFNO improved neither mechanical ventilatory assistance nor patient comfort compared with oxygen delivered via a Venturi mask. However, the study was underpowered because of the low event rate and the one-sided hypothesis.

**Trial registration:**

ClinicalTrials.gov identifier: NCT02424773. Registered 20 April 2015.

## Introduction

Acute respiratory failure (ARF) remains the most common and severe life-threatening complication in immunocompromised patients [1–3]. Hypoxemic ARF can be related to a variety of causes [4, 5], which must be identified by etiological investigations conducted simultaneously with symptomatic management. Many immunocompromised patients with ARF require ventilatory support within a few hours after admission to the intensive care unit (ICU) [6–8]. Avoiding invasive mechanical ventilation (IMV) significantly decreases the risk of death [1, 3]. Thus, choosing the optimal device for delivering oxygen is of the utmost importance to decrease the IMV rate while maintaining safe levels of oxygenation and ensuring patient comfort.

Noninvasive ventilation (NIV) has been reported to decrease mortality in immunocompromised patients with hypoxemic ARF [9]. However, NIV had smaller benefits in the most recent studies, chiefly because of a sharp drop in mortality among patients given IMV [10–13]. Moreover, NIV failure was associated with high mortality [14, 15] and with patient discomfort and anxiety [16].

High-flow nasal oxygen (HFNO) therapy was first introduced to treat children [17, 18], but it is now increasingly used in adults managed in emergency departments or ICUs [19–22]. This method consists of delivering a high flow of humidified oxygen through a large nasal cannula (HFNC). It not only delivers a high fraction of inspired oxygen (FiO_2_) but also generates some measure of positive pressure [23], ensures washout of the nasopharyngeal dead space [23, 24], and diminishes the work of breathing [25].

Few studies in adults have compared HFNO with oxygen delivered through a facemask. Most of them were small, uncontrolled, and used physiologic parameters as the primary endpoints. They showed improvements in oxygen saturation, partial pressure of arterial oxygen (PaO_2_), patient comfort, respiratory rate, and dyspnea [19, 21, 26, 27]. None compared intubation rates between the two oxygen delivery methods. A retrospective, uncontrolled study of 45 immunocompromised patients treated with HFNO found a 66 % intubation rate [28].

In the present study, our objective was to compare HFNO with oxygen delivered through a Venturi mask in immunocompromised patients admitted to the ICU with ARF. We conducted a multicenter randomized controlled trial with the primary endpoint of need for NIV or IMV within the first 2 h of oxygen therapy.

## Methods

### Overview

We performed an open, prospective, multicenter, parallel-group randomized controlled trial in four ICUs between November 2012 and April 2014. The appropriate ethics committee approved the research protocol (Comité de protection des personnes Ile de France IX, 12 November 2011, RCB 2011-A00241-40). Informed nonopposition consent was obtained from all patients before study inclusion. The funding source (Fisher & Paykel Healthcare, Auckland, NZ) provided the nasal cannulas for HFNO and funds for insurance but had no other role in the study.

### Patients

Consecutive immunocompromised patients admitted to the ICU for ARF were screened for inclusion. ARF was defined as onset of respiratory symptoms within 72 h before ICU admission and either a need for oxygen greater than 6 L/min to maintain peripheral capillary oxygen saturation (SpO_2_) above 95 % or symptoms of respiratory distress (tachypnea >30/min, intercostal recession, labored breathing, and/or dyspnea at rest). In addition to ICU admission for ARF, inclusion criteria were age over 18 years and immunosuppression (solid or hematological malignancy, solid organ transplant, long-term or high-dose [≥1 mg/kg/day] steroid therapy, other immunosuppressive treatment, or HIV infection). Exclusion criteria were hypercapnia (>45 mmHg), mechanical ventilation before ICU admission, need for immediate NIV or IMV, and patient refusal to participate in the study. Patients who met all inclusion criteria and none of the exclusion criteria were allocated at random in a 1:1 ratio, with stratification on study center, to HFNO or oxygen delivery via a Venturi mask with the use of a permuted block method. Opaque, sealed envelopes ensured identity concealment. The physician who included the patient opened the sealed envelope and started the oxygen device of the randomized group. The nature of the study treatments precluded blinding.

### Study treatments

Oxygen therapy was started immediately after inclusion. In the Venturi mask group, FiO_2_ was 60 % (15 L/min) initially and was then adjusted as needed to maintain SpO_2_ of at least 95 %. Humidification was not applied. In the HFNO group, HFNO was used with heated humidified circuit, and initial flow was 40–50 L/min with an FiO_2_ of 100 % and was then adjusted as needed to maintain SpO_2_ of at least 95 %. Respiratory deterioration in immunocompromised patients with ARF occurs within the first few hours after ICU admission; we therefore confined the study to the first 2 h of HFNO or Venturi mask oxygen delivery. No crossover between the two treatments was allowed during this time window.

### Data collection

The data were collected prospectively. The Simplified Acute Physiology Score II (SAPS II) was computed within 24 h of ICU admission, and the Sequential Organ Failure Assessment (SOFA) score was recorded at inclusion [29, 30]. At randomization and after 60 and 120 minutes of study treatment, we recorded the respiratory rate, heart rate, arterial blood pressure, SpO_2_, and FiO_2_. At the same time points, the patients completed three visual analogue scales (VASs; 0–10 scoring) on which 0 indicated absence and 10 the highest possible levels of dyspnea, discomfort, and thirst, respectively. The cause of ARF was established on the basis of predefined criteria [6]. We recorded the need for NIV and/or throughout the ICU stay and the ICU length of stay.

### Endpoints

The primary endpoint was the need for IMV or NIV during or at the end of the 2-h study period. IMV or NIV was started in patients who met at least one of the following criteria: worsening respiratory distress, defined as SpO_2_ less than 92 %, respiratory rate more than 40 breaths/min or labored breathing, regardless of the oxygen flow rate; inability to maintain PaO_2_ greater than 65 mmHg with FiO_2_ greater than 0.6; and hemodynamic or neurologic deterioration. The choice between NIV and IMV was at the physician’s discretion.

Secondary endpoints were the VAS scores for comfort, thirst, and dyspnea; respiratory rate; and heart rate.

### Statistical analysis

The sample size calculation was based on superiority of the HFNC strategy. On the basis of an earlier study [31], we expected oxygenation failure rates of 30 % in the Venturi mask group and 10 % in the HFNC group at the end of the 2-h trial. A sample size of 49 in each group would have a power of 80 % to detect such a difference (with a one-sided α of 0.05). The total number of patients included was rounded up to 100.

All analyses regarding the primary outcome were performed on the basis of the intention-to-treat principle. We compared the proportion of patients with a primary endpoint (including analyses of the separate components, NIV or intubation and IMV) between the two strategies and tested for significance using Fisher’s exact test. Because the observed effect was in the opposite direction, the tests were two-sided to ensure the possibility of assessing the statistically significant superiority of the Venturi mask strategy. Secondary continuous endpoints (comfort, thirst, dyspnea, heart rate, and respiratory rate) were compared between the two strategies among patients still receiving the planned treatment at the end of the 2-h trial using the Wilcoxon rank-sum test. All statistical analyses were performed using R software (version 2.13.1).

## Results

**Fig. 1:**
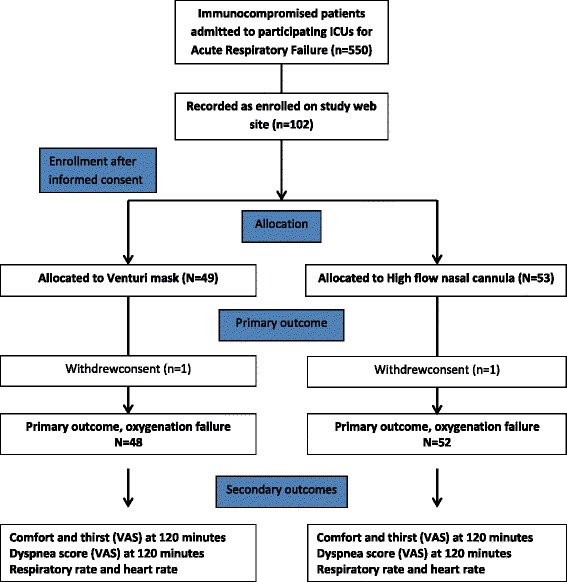
Patient flowchart. *HFNO* high-flow nasal oxygen, *ICU* intensive care unit, *IMV* invasive mechanical ventilation, *NIV* noninvasive ventilation, *VAS* visual analogue scale

Figure 1 is the patient flowchart. Of 550 consecutive immunocompromised patients admitted to the 4 participating ICUs, 102 met our selection criteria and were randomized: 53 to the HFNO group and 49 to the Venturi mask group. In each group, one patient withdrew consent. Thus, the final analysis included 100 patients: 52 in the HFNO group and 48 in the Venturi mask group.

### Patient characteristics

We report the baseline patient characteristics in Table 1. The most common causes of immunosuppression were steroid or immunosuppressant treatment (n = 65) and hematological malignancies (n = 61), and 15 % of patients had more than one cause of immunosuppression. At ICU admission, the patients had tachypnea (respiratory rate, 27 [22–32] breaths/min) and SpO_2_ of 96 % [94–98 %] under 12 [6–12] L/min of oxygen through a standard oxygen mask, a SOFA score of 3 [2–5], and an SAPS II of 39 [30–51]. The primary cause of ARF was related to sepsis for 50 patients (Table 1).

**Table 1:** Patient characteristics at randomization

Variables	HFNO group	Venturi mask group
	(n = 52)	(n = 48)
Age, yr, median [25th–75th percentile]	59.3 [43–70]	64.5 [53.25–72]
Males, n (%)	38 (73.1)	32 (66.7)
Comorbidities, n (%)		
Chronic respiratory failure	7 (13.5)	4 (8.3)
Chronic kidney failure	2 (3.8)	3 (6.2)
Chronic heart failure	4 (7.7)	2 (4.2)
Cause of immunosuppression, n (%)		
Solid malignancy	15 (28.8)	8 (16.7)
Hematological malignancy	31 (59.6)	30 (62.5)
HIV infection	3 (5.8)	5 (10.4)
Steroid treatment	13 (25.0)	15 (31.2)
Other immunosuppressant drugs	23 (44.2)	14 (29.2)
Final etiology of ARF,^b^ n (%)		
Sepsis	25 (48.1)	25 (52.0)
Cardiogenic pulmonary edema	5 (9.6)	2 (4.1)
Noninfectious pulmonary disease	5 (6.8)	7 (14.5)
Lung involvement by the underlying disease	7 (13.4)	9 (18.7)
Large pleural effusion	0 (0)	1 (2.0)
*Pneumocystis* pneumonia	5 (9.6)	2 (4.1)
Miscellaneous^c^	3 (5.7)	1 (2.0)
No diagnosis	2 (3.8 %)	1 (2.0)
SAPS II at ICU admission, median [25th–75th percentile]	42 [29.5–52]	37.5 [31.5–46.5]
SOFA score at randomization, median [25th–75th percentile]	3.5 [2–6]	3 [2–5]
Days since respiratory symptom onset	3 [2–8]	3 [2–7.25]
Clinical status at randomization		
Respiratory rate, breaths/min, median [25th-75th percentile]	26 [21.7–31.2]	27 [22–32.2]
SpO_2_, %, median [25th-75th percentile]	96 [94–98]	96 [95–98.2]
Estimated PaO_2_/FiO_2_ ratio at admission	128 [48–178]	100 [40–156]
Mean arterial pressure, mmHg, median [25th-75th percentile]	86.8 [82.2–95.3]	80 [74–89.3]
Normal Glasgow Coma Scale score, n (%)	49 (94.2)	48 (100)
Confusion, n (%)	4 (7.7)	1 (2.1)

*HFNO* high-flow nasal oxygen, *ARF* acute respiratory failure, *ICU* intensive care unit, *SAPS II* Simplified Acute Physiology Score II (range 0–163 points, with worse scores indicating greater disease severity), *SOFA* Sequential Organ Failure Assessment, *SpO*_*2*_ peripheral capillary oxygen saturation, *PaO*_*2*_ */FiO*_*2*_ ratio of partial pressure of arterial oxygen to fraction of inspired oxygen

^a^The groups were compared using the χ^2^ test for qualitative variables and the Wilcoxon test for quantitative variables.

^b^More than one etiology could be suspected at admission.

^c^Pulmonary embolism, lung metastasis, neutropenia recovery, extrapulmonary acute respiratory distress syndrome, drug-related pulmonary toxicity

**Fig. 2:**
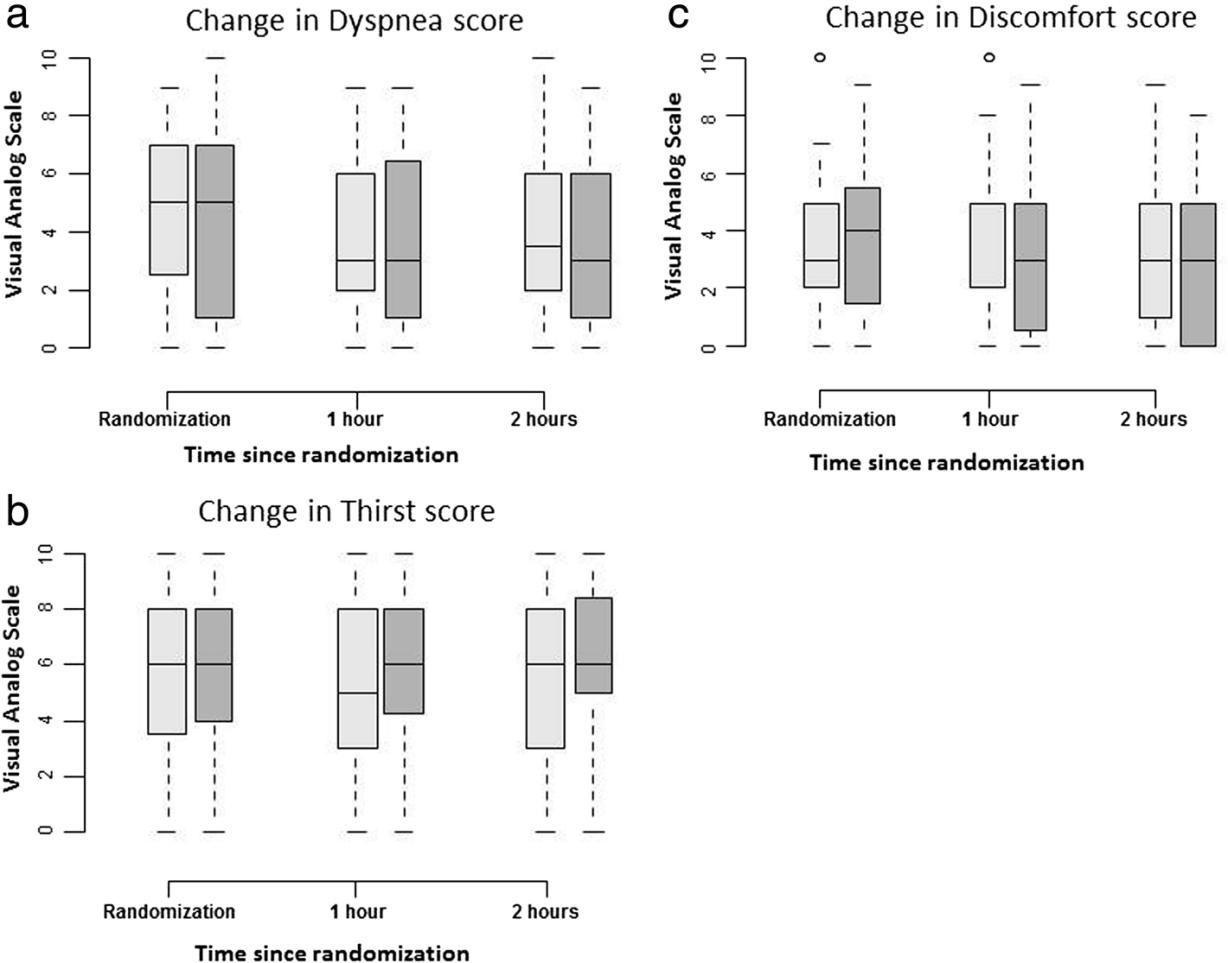
Changes in dyspnea, thirst and discomfort during the 2-h study period. Dyspnea (panel **a**), thirst (panel **b**) and discomfort (panel **c**) were assessed using 0–10 visual analogue scales on which 0 indicated absence of dyspnea, thirst, or discomfort and 10 signified the worst possible dyspnea, thirst, or discomfort. The *open bars* represent the high-flow nasal oxygen group and the *gray bars* the Venturi mask group

None of the patients received oxygen through a Venturi mask, HFNO, or NIV before randomization, which occurred on day 0 [0–1] after ICU admission. Before randomization, oxygen was provided through oxygen prong, oxygen bagless mask, or oxygen bag mask. At baseline, the VAS scores were 3 [2–5] for discomfort, 5 [2–7] for dyspnea, and 6 [4–8] for thirst (Table 2 and Fig. 2).

**Table 2:** Primary and secondary endpoints in the two treatment groups

	HFNO group	Venturi mask group	*P* value
	(n = 52)	(n = 48)	
Primary endpoint			
Number (%) of patients requiring mechanical ventilation	8 (15 %)	4 (8 %)	0.36
Noninvasive mechanical ventilation	6^a^	3^a^	
Invasive mechanical ventilation	4	2	
Secondary endpoints, median [25th–75th percentile]			
Discomfort VAS score^b^ at 120 min	3 [1–5]	3 [0–5]	0.88
Dyspnea VAS score^b^ at 120 min	3 [2 – 6]	3 [1–6]	0.87
Thirst VAS score^b^ at 120 min	6 [3–8]	6 [5 – 9]	0.40
Respiratory rate at 120 min, breaths/min	25 [22–29]	25 [21–31]	
Heart rate at 120 min, beats/min	98 [90–110]	99 [83–112]	0.43

*HFNO* high-flow nasal oxygen, *VAS* visual analogue scale

^a^Two patients in the HFNO group and one patient in the Venturi mask group received noninvasive ventilation followed by invasive mechanical ventilation

^b^All three VASs ranged from 0 (absence of discomfort, dyspnea, or thirst) to 10 (worst possible discomfort, dyspnea, or thirst)

### Study endpoints

Of the 100 patients, 88 received the randomly allocated oxygen treatment throughout the 2-h period and 12 required NIV or IMV before the end of the 2-h period. There was no significant difference between the HFNO and Venturi mask groups regarding the need for IMV/NIV during the 2-h study period (Table 2).

**Fig. 3:**
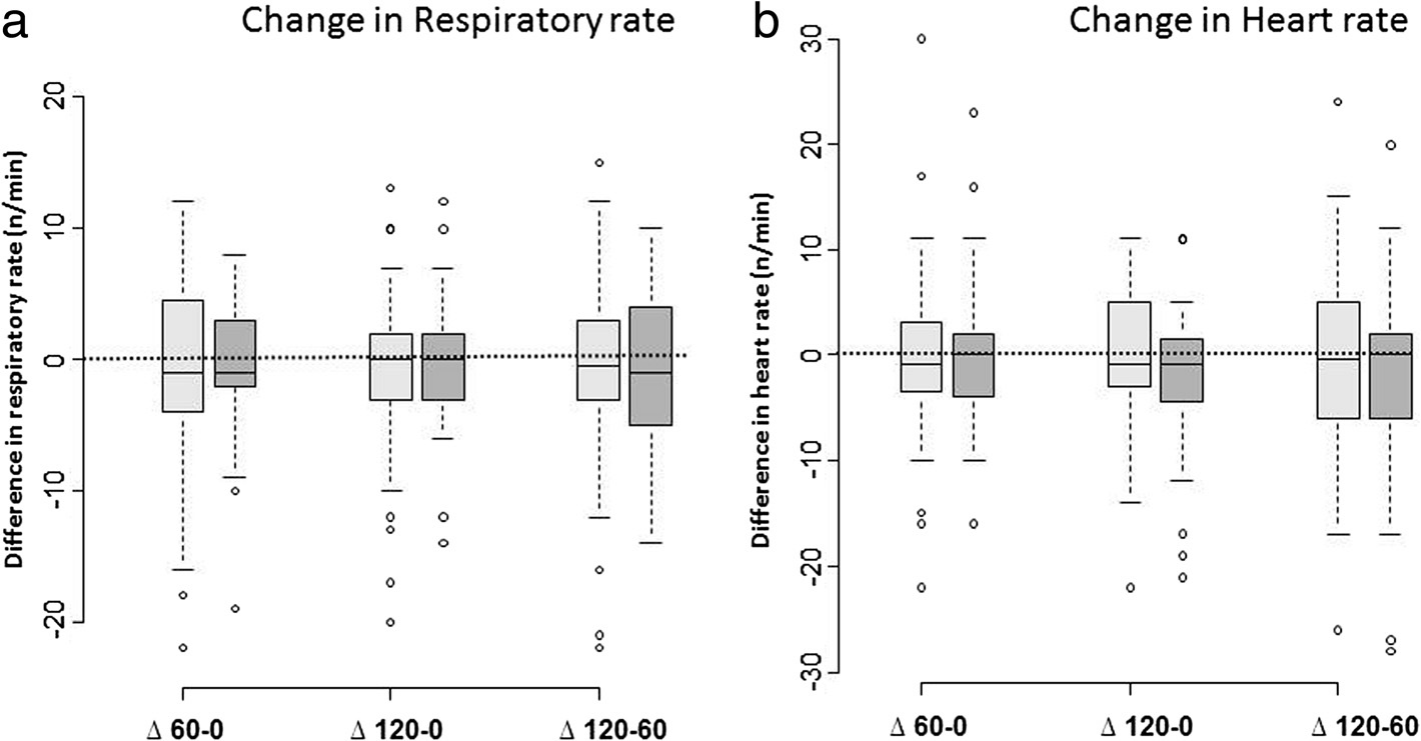
Respiratory rate (panel **a**) and heart rate (panel **b**) changes during the 2-h study period. The *open bars* represent the high-flow nasal oxygen group and the *gray bars* the Venturi mask group. *∆60-0* indicates the difference between randomization and the 1-h time point. *∆120-60* indicates the difference between the 1-h and 2-h time points. *∆120-0* indicates the difference between randomization and the 2-h time point

None of the secondary endpoints differed significantly between the Venturi mask and HFNO groups (Table 2 and Figs. 2 and 3). Moreover, we did not find an interaction between etiology of ARF (sepsis vs. no sepsis) and efficacy of oxygen device (*P* = 0.44).

### Subsequent outcomes

IMV was required within 1 day after randomization in three additional patients: one in the HFNO group and two in the Venturi mask group. Throughout their ICU stay, a total of 39 patients required IMV. In patients who required intubation after the end of the study period, IMV occurred within 1 [0–2] day. Oxygen was required for 5 [2–8] days. ICU length of stay was 7 [3–13] days, and ICU mortality was 24 %.

## Discussion

In this randomized trial in immunocompromised patients with ARF, mechanical ventilation requirements within the first 2 h showed no significant difference between HFNO and oxygen delivered via a Venturi mask. In keeping with this finding, no differences were found for respiratory rate, dyspnea score, or heart rate. Finally, patient comfort was not different between the two treatments.

A randomized trial with a final crossover period compared HFNO with standard nonhumidified oxygen therapy in 37 consecutive ICU patients with ARF who did not require immediate NIV or MIV [32]. Dryness and discomfort were significantly lower in the HFNO group. The patients were not immunocompromised, and they had less severe hypoxemia compared with those in our study. In a trial conducted in a cardiothoracic and vascular ICU, researchers randomized 60 patients to HFNO or heated and humidified oxygen therapy via a standard facemask [31]. The need for IMV or NIV within 24 h was lower in the HFNO group (10.3 % vs. 30 %, *P* = 0.006). Neither comfort nor dyspnea was assessed. Moreover, most of the patients had undergone heart surgery and were therefore likely to benefit from the positive expiratory pressure delivered by HFNO [31]. Our patients had severe hypoxemia and immune deficiencies. In a randomized trial with 310 patients with hypoxemic ARF, the intubation rate was lower with HFNO than with NIV or standard oxygen in the subgroup with the most severe hypoxemia at baseline (PaO_2_/FiO_2_ ratio ≤200), but not in the overall population [33]. In the FLORALI study, 26 % of patients were immunocompromised. Among patients with ARF, those with hematological malignancies more often require NIV or IMV than other patients do [22, 31]. Thus, of 45 patients with hematological malignancies who received HFNO for ARF, 30 (66 %) required IMV [28].

The failure of HFNO to decrease the need for mechanical ventilatory assistance in our study may be ascribable to several factors. The underlying disease associated with immune deficiency (e.g., malignancy, transplantation, or systemic inflammatory disease) may have been a source of patient discomfort that was not influenced by the mode of oxygen delivery. Second, the time needed to improve oxygenation during ARF may be longer in immunocompromised patients than in other patients [34]. Thus, a longer trial would perhaps have provided different results. However, respiratory deterioration in patients with malignancies has been reported to occur chiefly within a few hours of ARF onset [6]. Third, although researchers in most studies of HFNO in adults used a 40 L/min oxygen flow [19, 22, 23], as in our trial, there is some evidence that higher flows may improve comfort by increasing air humidification [25, 35]. Investigators in a crossover study compared HFNO, oxygen via a Venturi mask, and continuous positive airway pressure in ten ICU patients immediately after tracheostomy removal [23]. Each device was tested with three oxygen flow rates (15, 30, and 45 L/min). However, in that study as in our study, HFNO did not improve patient comfort compared with the Venturi mask. Fourth, in our 2-h study, NIV was not administered before randomization. In a randomized trial of postextubation HFNO versus standard care in 340 heart surgery patients, although the oxygenation failure rate was higher with standard care, comfort scores were lower with HFNO [36].

The present study has several limitations. We did not use a crossover design. However, ARF can worsen quickly in immunocompromised patients, confounding the assessment of patient comfort, and we therefore limited the study period to 2 h [37]. HFNO patients remained on HFNO throughout the 2-h study. Second, sources of discomfort were not assessed. Discomfort can be due to dyspnea, HFNO-related noise, nasal obstruction, or sources unrelated to ARF or its treatment. In a previous study, HFNO compared with a standard oxygen mask did not significantly diminish nasal obstruction but did improve comfort by decreasing dryness [32]. Moreover, in a recent study, airway dryness was different in the two groups after 24 h. A longer study would allow different results. A new HFNO device that is less noisy has been developed but was not evaluated in our study. Third, the low frequency of HFNO failure precluded a statistical assessment of factors independently associated with IMV or NIV. Identifying such factors would be of interest, as prolonged HFNO therapy might worsen outcomes by delaying mechanical ventilation [37, 38]. Fourth, the statistical power of our study was lower than planned, as the oxygen failure rate in the Venturi mask group was 8 % instead of the expected 25 %. Moreover, the study was initially designed to demonstrate superiority of HFNC using a one-sided test; thus, it was clearly underpowered to demonstrate the inferiority of HFNC compared with the Venturi facial oxygen mask. At the time the study protocol was developed, the number of patients needed was assessed on the basis of Parke et al.’s study, which was the only published study in which intubation rate was compared between the two devices. In the Parke et al. 's study, HFNO failure was assessed within 24 h in cardiovascular patients. However, ARF in immunocompromised patients could worsen faster and intubation could occur earlier than in cardiovascular patients. For this reason, we did not perform a crossover study. Within the short duration of our study, we recorded a low failure rate. Patients intubated after the study period required IMV within 1 [0–2] day. A longer study period would produce different results. Moreover, patients were admitted and included in the study very early, without any other organ failure, but they were severely hypoxemic and 39 % of them were ultimately intubated.

## Conclusions

In immunocompromised patients with ARF, 2 h of HFNO neither decreased the need for mechanical ventilation nor improved patient comfort. However, the study was underpowered because of the low event rate and one-sided hypothesis. Studies aimed at identifying the sources of discomfort and targets for improvement are needed, as are longer trials of HFNO in immunocompromised patients with ARF.

## Key messages

- In immunocompromised patients admitted to the ICU with acute respiratory failure, the need for noninvasive or invasive mechanical ventilation within the first 2 h was not significantly different between the group given high-flow nasal oxygen therapy and the group given oxygen via a Venturi mask.
- No differences were found between the two groups with regard to patient comfort, dyspnea, respiratory rate, or heart rate.

## Abbreviations

ARF: Acute respiratory failure
FiO_2_: Fraction of inspired oxygen
HFNC: High-flow nasal cannula
HFNO: High-flow nasal oxygen
ICU: Intensive care unit
IMV: Invasive mechanical ventilation
NIV: Noninvasive ventilation
PaO_2_: Partial pressure of arterial oxygen
SAPS: Simplified Acute Physiology Score
SOFA: Sequential Organ Failure Assessment
SpO_2_: Peripheral capillary oxygen saturation
VAS: Visual analogue scale
